# Safety and feasibility of minimally invasive surgical interventions for esophageal and gastric cancer in the acute setting: a nationwide cohort study

**DOI:** 10.1007/s00464-020-07491-x

**Published:** 2020-03-25

**Authors:** Alicia S. Borggreve, B. Feike Kingma, Jelle P. Ruurda, Richard van Hillegersberg, the Dutch Upper G.I. Cancer Audit (DUCA) group

**Affiliations:** 1 Department of Surgery, University Medical Center Utrecht, Utrecht University, Heidelberglaan 100, 3584 CX Utrecht, The Netherlands; 2 Department of Radiation Oncology, University Medical Center Utrecht, Utrecht University, Heidelberglaan 100, 3584 CX Utrecht, The Netherlands

**Keywords:** Esophageal cancer, Gastric cancer, Acute surgery, Minimally invasive surgery, Open surgery, Postoperative complications, Oncological outcomes

## Abstract

**Background:**

Minimally invasive esophagectomy and gastrectomy are increasingly performed and might be superior to their open equivalents in an elective setting. The aim of this study was to evaluate whether minimally invasive approaches can be safely applied in the acute setting as well.

**Methods:**

All patients who underwent an acute surgical intervention for primary esophageal or gastric cancer between 2011 and 2017 were identified from the nationwide database of the Dutch Upper GI Cancer Audit (DUCA). Conversion rates, postoperative complications, re-interventions, postoperative mortality, hospital stay and oncological outcomes (radical resection rates and median lymph node yield) were evaluated.

**Results:**

Between 2011 and 2017, surgery for esophagogastric cancer was performed in an acute setting in 2% (190/8861) in The Netherlands. A total of 14 acute resections for esophageal cancer were performed, which included 7 minimally invasive esophagectomies and 7 open esophagectomies. As these numbers were very low, no comparison between minimally invasive and open esophagectomies was made. A total of 122 acute resections for gastric cancer were performed, which included 39 minimally invasive gastrectomies and 83 open gastrectomies. Conversion occurred in 9 patients (23%). Minimally invasive gastrectomy was at least comparable to open gastrectomy regarding postoperative complications (36% versus 51%), median hospital stay (9 days [IQR: 7–16 days] versus 11 days [IQR: 7–17 days]), readmissions (8% versus 11%) and oncological outcomes (radical resection rate: 87% versus 66%, median lymph node yield: 21 [IQR: 15–32 days] versus 16 [IQR: 11–24 days]).

**Conclusions:**

Minimally invasive surgery for gastric cancer is safe and feasible in the acute setting, with at least comparable postoperative clinical and short-term oncological outcomes compared to open surgery but a relatively high conversion rate.

Minimally invasive surgical techniques are increasingly being applied in the surgical treatment of esophageal and gastric cancer [1, 2]. Evidence from randomized controlled trials and nationwide studies suggests that these techniques might provide benefits over the traditional open approaches, especially regarding short-term outcomes in terms of postoperative morbidity and length of hospital stay [3–12]. In contrast, higher reintervention rates were observed after minimally invasive esophagectomies in population-based studies [5–8]. As the aforementioned studies only included patients who underwent an elective surgical resection, the generalizability of these results to the acute setting might be limited.

Acute surgery for esophageal and gastric cancer is relatively rare and is usually only performed in case tumors are actively bleeding or have perforated. These cases are different from the usual elective patient population and might therefore be more difficult to treat with minimally invasive surgical techniques. More research is warranted to investigate the role of minimally invasive surgical techniques for patients with esophageal and gastric cancer who have an acute indication for a resection. Therefore, the aim of this nationwide cohort study was to describe the postoperative outcomes of minimally invasive as compared to open acute surgery for esophageal and gastric cancer.

## Methods

### Study design

This nationwide observational cohort study was conducted with data from the Dutch Upper GI Cancer Audit (DUCA), a nationwide registration of all patients undergoing surgery for esophageal and gastric cancer since 2011 [13]. Registration of cases in the DUCA is mandatory for each hospital and includes patient characteristics, treatment details including the timing of surgery, postoperative outcomes (until 30 days after surgery), and pathological outcomes. The scientific committee of the DICA and DUCA approved this study. No ethical approval or informed consent was required under Dutch law.

### Study population and treatment

All patients who underwent acute surgery with the intention to perform an esophagectomy or gastrectomy for cancer between 2011 and 2017 were selected from the DUCA registry. Patients were diagnosed according to the Dutch national guidelines for diagnosis, treatment, follow up, and guidance of patients for patients with esophageal and gastric cancer [14, 15]. Acute surgical interventions were either defined as emergent (i.e., surgery scheduled < 12 h after presentation with an acute indication) or urgent (surgery scheduled > 12 h—but not electively—after presentation with an acute indication). Both were included in the current study. Exclusion criteria were prophylactic surgical indications (i.e., no proven malignancy), insufficient data regarding the tumor or surgical approach, and surgery for cancer recurrence.

### Outcome measures

The outcome measures included the rates of conversion to an open procedure, postoperative complications, re-interventions, postoperative mortality (i.e., mortality during initial hospital admission or within 30 days after surgery), length of postoperative stay on the Intensive Care Unit (ICU) and in hospital, as well as readmissions (< 30 days after discharge). Complications were defined according to standards of the DUCA, and included pulmonary complications (clinically proven pneumonia, pleural effusion leading to drainage, pleural empyema and/or acute respiratory distress syndrome), anastomotic leakage (either by clinical or radiological diagnosis), cardiac complications (supraventricular and ventricular arrhythmia, myocardial infarction and/or heart failure), thromboembolic complications (pulmonary embolism, deep venous thrombosis, stroke and/or thrombophlebitis), neurologic complications (recurrent laryngeal nerve injury and/or acute delirium), urologic complications (urinary tract infection, urinary retention and/or renal insufficiency), intra-abdominal abscess, chyle leakage, fascia dehiscence and wound infections. Furthermore, oncological outcomes in terms of radicality and total lymph node yield were analyzed. A radical resection (i.e., R0) was defined as the absence of tumor cells within the resection margins of the resection specimen.

### Statistical analyses

Patient and treatment characteristics were described as counts with percentages, mean (± standard deviation [SD]) or median (interquartile range [IQR]). The postoperative outcomes were separately described for patients who underwent minimally invasive surgery and open surgery. No tests for statistical significance of differences between groups were performed because of inability to adequately correct for confounding bias due to small group sizes. Statistical analyses were performed using SPSS 23.0 (IBM Corp., Armonk, NY, USA).

## Results

### Study population

**Fig. 1:**
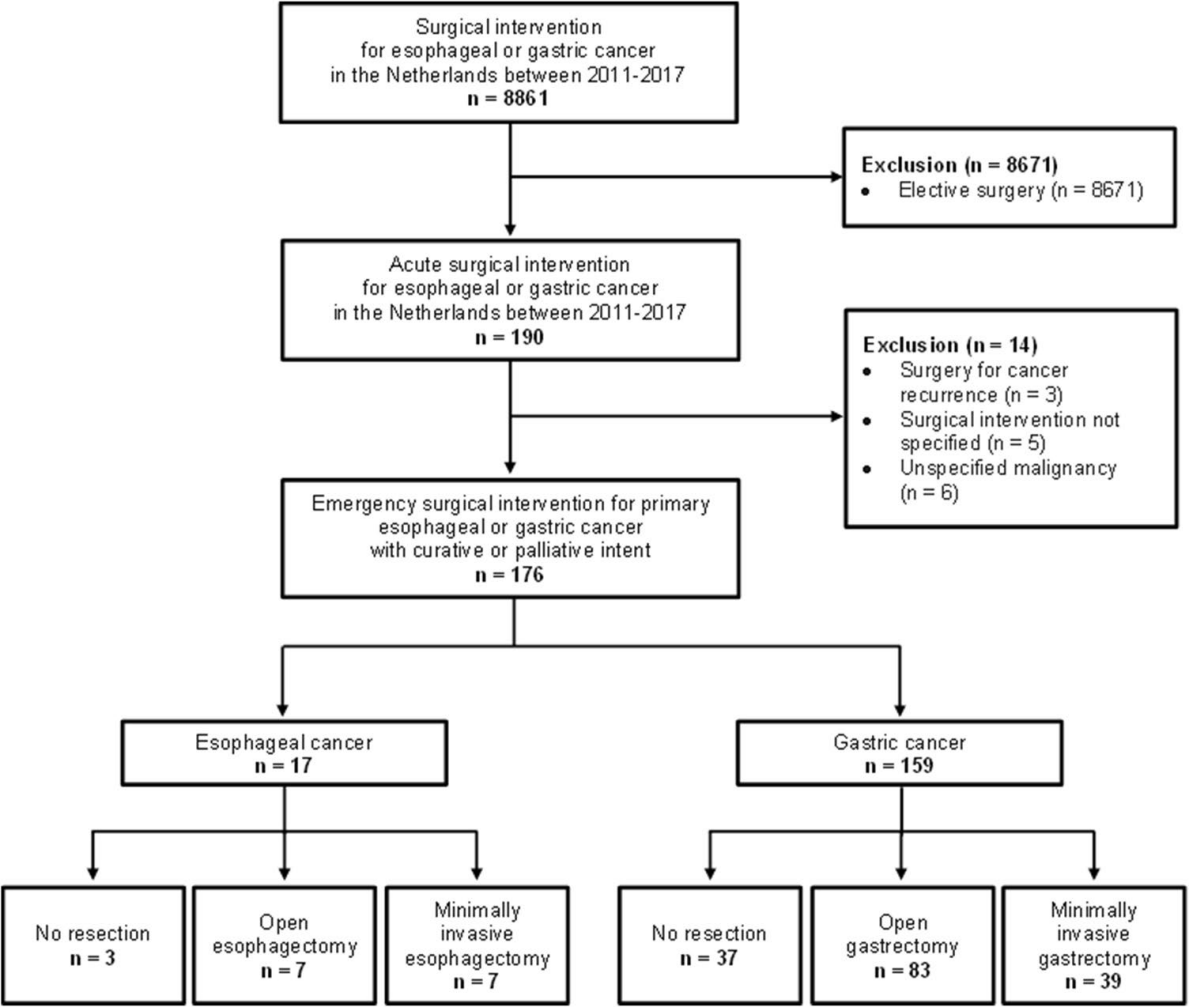
Study flowchart

Between 2011 and 2017, a total of 8861 patients who underwent surgery for esophageal or gastric cancer in the Netherlands were registered in the DUCA. Surgery was performed in an acute setting in 190 out of these 8861 patients (2%). Patients who underwent an unspecified surgical intervention (*n* = 5), a surgical procedure for cancer recurrence (*n* = 3), or a surgical procedure for an unspecified malignancy (*n* = 6) were excluded. Hence, a total of 176 patients were included, of whom 17 patients with esophageal cancer and 159 patients with gastric cancer (Fig. 1).

### Acute surgical interventions for esophageal cancer

Acute surgical interventions for esophageal cancer were performed in 17 patients and were with curative intent in all of these patients. The median number of days between the diagnostic biopsy and surgery was 70 days (IQR 46–121 days). In 2 patients (12%), no histologic diagnosis was present prior to surgery. The reason for surgery was a perforation in 7 patients (41%), bleeding in 5 patients (29%) and other (not specified) in the remaining 5 cases (29%). Esophagectomy was performed in 14 out of these 17 patients (82%); by a minimally invasive transhiatal approach in 4 patients, an open transhiatal approach in 5 patients, a minimally invasive transthoracic approach in 3 patients, and an open transthoracic approach in 2 patients. The remaining 3 patients (18%) underwent a surgical procedure without resection, which involved a diagnostic laparoscopy in 2 patients and a diagnostic thoracotomy in 1 patient. A complete overview of patient and treatment characteristics of the esophageal cancer patients is presented in Table 1.

**Table 1:** Baseline characteristics of patients who underwent an acute surgical intervention for esophageal cancer in the Netherlands between 2011 and 2017

Characteristics	All	
	*n* = 17	%
Patient-related characteristics		
Age, years (mean ± SD)	64 ± 11	
Sex		
Male	15	88%
Female	2	12%
BMI, kg/m^2^ (mean ± SD)	26 ± 5	
ASA classification		
I	2	12%
II	7	41%
III	7	41%
IV	1	6%
Comorbidities		
Cardiac	2	12%
Vascular	8	47%
Diabetes	5	29%
Pulmonary	3	18%
Previous abdominal or thoracic surgery	7	41%
Tumor location		
Middle esophagus	1	6%
Distal esophagus	9	53%
Gastro-esophageal junction	5	29%
Unknown	2	12%
Histology		
Adenocarcinoma	15	88%
Squamous cell carcinoma	1	6%
Other	1	6%
cT status^a^		
T1	3	18%
T2	2	12%
T3	5	29%
T4	5	29%
Tx	2	12%
cN status^a^		
N0	6	35%
N+	8	47%
Nx	3	18%
cM status^a^		
M0	13	76%
M1	2	12%
Mx	2	12%
Treatment-related characteristics		
Neoadjuvant therapy^b^		
None	10	59%
Chemoradiotherapy	4	24%
Chemotherapy	2	12%
Radiotherapy	1	6%
Setting		
Emergent (< 12 h)	10	59%
Urgent (> 12 h)	8	41%
Reason for emergency surgery		
Bleeding	5	29%
Perforation	7	41%
Other/unknown	5	29%
Surgical approach		
Minimally invasive	9	53%
Open	8	47%
Surgical procedure		
Transhiatal esophagectomy	9	53%
Transthoracic esophagectomy	5	29%
Diagnostic thoracotomy	1	6%
Diagnostic laparoscopy	2	12%
Reconstruction		
Gastric conduit reconstruction	12	71%
No reconstruction	4	24%
Unknown	1	6%
Location of anastomosis		
Cervical	10	59%
Intrathoracic	2	12%
Not applicable	5	29%
Lymph node dissection	13	76%
Year of surgery		
2011–2013	9	53%
2014–2017	8	47%

Data are numbers of patients with column-based percentages in parentheses, unless otherwise stated

*ASA* American Society of Anesthesiologists*; BMI* body mass index at diagnosis; *SD* standard deviation

^a^Clinical T status and N status are based on AJCC TNM 7th edition

^b^The standard regimen for neoadjuvant treatment for esophageal cancer patients in the Netherlands consists of carboplatin and paclitaxel, weekly during 5 weeks, and concurrent radiotherapy with a total radiation dose of 41.4 Gy in 23 fractions of 1.8 Gy. For gastro-esophageal junction or gastric adenocarcinoma, peri-operative treatment generally consists of chemotherapy regimens similar to the MAGIC-trial (epirubicin, cisplatin and capecitabine)

Perioperative and oncological outcomes of the 14 patients who underwent esophagectomy are shown in Table 2. The outcomes were not reported for minimally invasive esophagectomy and open esophagectomy separately, as the number of patients for both groups was only 7. Postoperative complications occurred in 50% (7/14). Pulmonary complications were most common (43%, 6/14), followed by anastomotic leakage (29%, 4/14). The median length of hospital stay was 13 days (IQR: 11–26 days). No patients were readmitted to the hospital within 30 days after discharge.

**Table 2:** Short-term outcomes for patients who underwent an acute esophagectomy for esophageal cancer in the Netherlands between 2011 and 2017

	Total	
	*n* = 14	%
Peroperative outcomes		
Conversion^a^	1	7%
Postoperative complications		
All	7	50%
Pulmonary^b^	6	43%
Anastomotic leakage^c^	4	29%
Cardiac^d^	1	7%
Chyle leakage	0	0%
Re-interventions	1^g^	7%
Recovery		
ICU duration (median, IQR)	3	(1–6)
Length of stay (median, IQR)	13	(11–26)
Postoperative mortality^e^	1	7%
Readmission to hospital^f^	0	0%
Pathological outcomes		
Radicality		
R0	9	64%
R1	5	36%
Lymph node yield (median, IQR)	21	(12–26)
Positive lymph nodes harvested (median, IQR)	3	(1–10)

There were no missing values for the variables described in this table

*IQR* interquartile range, *NA* not applicable

^a^Conversion > 30 min after start of surgery because of peroperative bleeding

^b^Pneumonia, pleural effusion, respiratory failure, pneumothorax and/or acute respiratory distress syndrome

^c^Any clinically or radiologically proven anastomotic leakage

^d^Supraventricular and ventricular arrhythmia, myocardial infarction and/or heart failure

^e^Death during initial hospital admission or within 30 days after surgery

^f^Readmission to hospital within 30 days after initial discharge

^g^Re-operation for anastomotic leakage

Histopathological evaluation of the resection specimen showed that a radical resection was achieved in 64% (9/14). The median lymph node yield was 21 (IQR: 12–26 days).

### Acute surgical interventions for gastric cancer

**Fig. 2:**
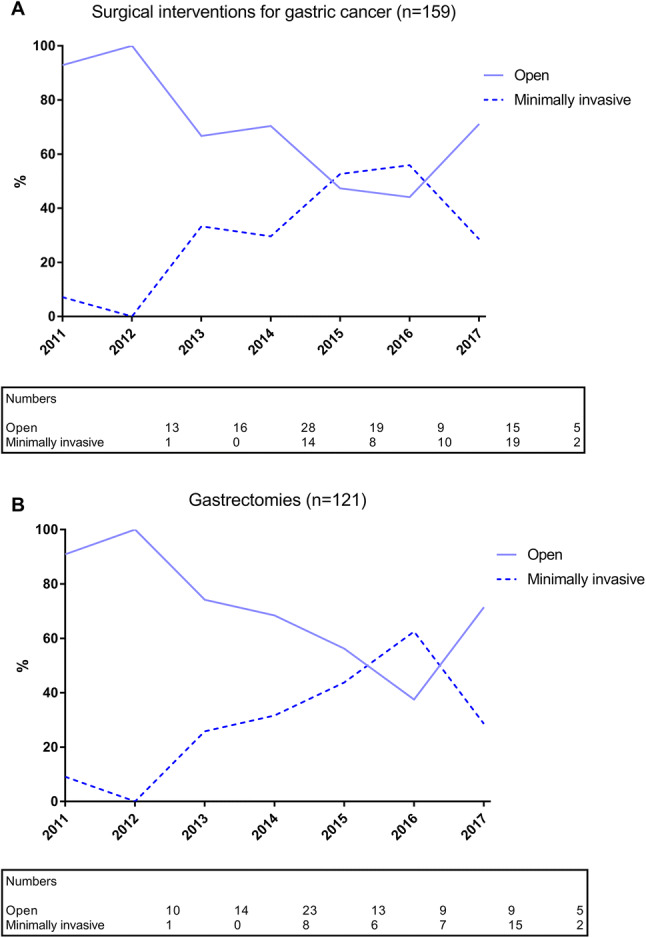
Minimally invasive and open surgical interventions (**A**) and gastrectomies (**B**) for acute presentation of gastric cancer in the study period

An acute surgical intervention for gastric cancer was performed in 159 patients, with an upfront curative intent in the majority of patients (70%). The median time between diagnostic biopsy and acute surgery was 23 days (IQR 11–36 days). Histological confirmation of gastric cancer was not present prior to surgery in 25 patients (16%). The reason for surgery was a bleeding in 68 patients (43%), perforation in 19 patients (12%) and other (not specified) in the remaining 73 patients (46%). Gastrectomy was performed in 122 patients (77%), which was by a minimally invasive approach in 39 patients and by an open approach in 83 patients. The remaining 37 patients (23%) underwent a surgical procedure without resection, which involved a gastroenterostomy in 26 patients, diagnostic laparoscopy in 6 patients and a diagnostic laparotomy in 5 patients. Most minimally invasive procedures were observed in the more recent years (Fig. 2). A complete overview of patient and treatment-related characteristics of the gastric cancer patients is presented in Table 3.

**Table 3:** Baseline characteristics of patients who underwent an acute surgical intervention for gastric cancer in the Netherlands between 2011 and 2017

Characteristics	Open		Minimally invasive	
	*n* = 105	%	*n* = 54	%
Patient-related characteristics				
Age, years (mean ± SD)	71 ± 10		71 ± 12	
Sex				
Male	79	75	33	61
Female	26	25	21	39
BMI, kg/m^2^ (mean ± SD)	25 ± 4		25 ± 4	
ASA classification				
I	8	8	2	4
II	39	37	25	46
III	46	44	23	43
IV	7	7	4	7
V	1	1	0	0
Not specified	4	4	0	0
Comorbidities				
Cardiac	35	33	24	44
Vascular	42	40	27	50
Diabetes	22	21	10	19
Pulmonary	23	22	11	20
Previous abdominal or thoracic surgery	37	35	18	33
Tumor location				
Fundus	6	6	3	6
Corpus	28	27	12	22
Antrum	48	46%	22	41
Pylorus	12	11	12	22
Stomach	3	3	2	4
Other/not specified	8	8	3	6
Histology				
Adenocarcinoma	88	84	49	91
Other	8	8	4	7
Not specified	9	8	1	2
cT status^a^				
T1	4	4	2	4
T2	8	8	8	15
T3	32	30	20	37
T4	18	17	10	19
Tx	43	41	13	24
cN status^a^				
N0	26	25	19	35
N+	46	44	28	52
Nx	33	31	7	13
cM status^a^				
M0	76	72	44	81
M1	9	9	4	7
Mx	20	19	6	11
Treatment-related characteristics				
Neoadjuvant therapy^b^				
None	93	89	52	96
Chemotherapy	11	10	2	4
Chemoradiotherapy	1	1	0	0
Setting				
Emergent (< 12 h)	37	35	8	15
Urgent (> 12 h)	68	65	46	85
Reason for acute surgery				
Bleeding	41	39%	26	48
Perforation	17	16%	2	4
Other	47	45%	26	48
Surgical procedure				
Total gastrectomy	26	25%	8	15
Partial gastrectomy	57	54%	31	57
Bypass (gastroenterostomy)	17	16%	9	17
Diagnostic laparotomy/laparoscopy	5	5%	6	11
Intention				
Curative intent	74	70%	43	80
Palliative intent	26	25%	8	15
Not specified	5	5%	3	6
Lymph node dissection	64	61%	35	65
Year of surgery				
2011–2013	57	54%	15	28
2014–2017	48	46%	39	72

Data are numbers of patients with column-based percentages in parentheses, unless otherwise stated

*ASA* American Society of Anesthesiologists*; BMI* body mass index at diagnosis; *SD* standard deviation

^a^Clinical T status and N status are based on AJCC TNM 7th edition

^b^The standard regimen for peri-operative treatment for gastro-esophageal junction or gastric adenocarcinoma generally consists of chemotherapy regimens similar to the MAGIC-trial (epirubicin, cisplatin and capecitabine)

Perioperative and oncological outcomes of the 122 patients who underwent a gastrectomy are shown in Table 4. Conversion to an open procedure occurred in 9 of the 39 minimally invasive gastrectomies (23%), mostly because of the extent of the tumor and poor exposure (Table 4). Postoperative complications occurred in 14 of the 39 patients who underwent a minimally invasive gastrectomy (36%) and in 42 of the 83 patients who underwent an open gastrectomy (51%). When comparing minimally invasive gastrectomy versus open gastrectomy, pulmonary complications occurred in 13% versus 18%, anastomotic leakage in 8% versus 8%, and wound infection in 0% versus 13%, respectively. Re-interventions, mostly for anastomotic leakage in both groups, were performed in 23% after a minimally invasive gastrectomy versus 14% after an open gastrectomy. Postoperative mortality occurred in 8% after a minimally invasive gastrectomy versus 11% after an open gastrectomy. The median length of hospital stay was 9 days (IQR: 7–16 days) after minimally invasive gastrectomy versus 11 days (IQR: 7–17 days) after open gastrectomy. Readmission to the hospital was seen in 8% after minimally invasive gastrectomy versus 11% after open gastrectomy.

**Table 4:** Short-term outcomes for patients who underwent an acute gastrectomy for gastric cancer in the Netherlands between 2011 and 2017

	Open			Minimally invasive			
	*n* = 83	%		*n* = 39		%	
Peroperative outcomes							
Conversion	NA			9		23%	
Reasons for conversion	NA						
Tumor extent				4		10%	
Poor exposure				4		10%	
Peroperative complication				1		3%	
Postoperative complications							
All^a^	42	51%		14		36%	
Medical complications							
Pulmonary^b^	15	18%		5		13%	
Cardiac^c^	7	8%		2		5%	
Thromboembolic^d^	4	5%		2		5%	
Neurologic^e^	4	5%		2		5%	
Urologic^f^	4	5%		1		3%	
Intra-abdominal complications							
Anastomotic leakage^g^	7	8%		3		8%	
Abscess	4	5%		1		3%	
Chyle leakage	0	0%		0		0%	
Wound complications							
Fascia dehiscence	2	2%		0		0%	
Wound infection^h^	11	13%		0		0%	
Other	11	13%		5		5%	
Re-interventions							
All^a^	12	14%		9		23%	
Surgical	11	13%		6		15%	
Radiologic	3	4%		2		5%	
Endoscopic	2	2%		1		3%	
Not specified	0	0%		1^i^		3%	
Causes for re-interventions							
Anastomotic leakage	5	6%		2		5%	
Fascia dehiscence	1	1%		0		0%	
Complication of feeding jejunostomy	0	0%		1		3%	
No complication encountered	3	4%		2		5%	
Other	3	4%		3		8%	
Recovery							
ICU duration (median, IQR)	1	(0–2)		1		(0–1)	
Length of stay (median, IQR)	11	(7–17)		9		(7–16)	
Postoperative mortality^j^	9	11%		3		8%	
Readmission to hospital^k^	9	11%		3		8%	
Oncological outcomes							
Radicality							
R0	55	66%		34		87%	
R1	14	17%		3		8%	
R2	11	13%		0		0%	
Lymph node yield (median, IQR)	16	(11–24)		21		(15–32)	
Positive lymph nodes harvested (median, IQR)	4	(0–9)		5		(1–10)	

*IQR* interquartile range, *NA* not applicable

Missing values were encountered in ICU duration (*n* = 12), length of stay (*n* = 2), readmission (*n* = 4), radicality (*n* = 5), lymph node yield (*n* = 2) and positive lymph nodes harvested (*n* = 2)

^a^Defined as number of patients who experienced a postoperative complication or underwent a reintervention. One patient could experience more than 1 complication or reintervention

^b^Pneumonia, pleural effusion, respiratory failure, pneumothorax and/or acute respiratory distress syndrome

^c^Supraventricular and ventricular arrhythmia, myocardial infarction and/or heart failure

^d^Pulmonary embolism, deep venous thrombosis, stroke and/or thrombophlebitis

^e^Acute delirium

^f^Urinary tract infection, urinary retention and/or renal insufficiency

^g^Any clinically or radiologically proven anastomotic leakage

^h^Wound infection requiring drainage or antibiotic treatment

^i^Patient who underwent a reintervention for a complication of the feeding jejunostomy

^j^Death during initial hospital admission or within 30 days after surgery

^k^Readmission to hospital within 30 days after initial discharge

Histopathological evaluation of the resection specimen showed that a radical resection was achieved in 34 of the minimally invasive gastrectomies (87%) and in 55 of the open gastrectomies (66%). The median lymph node yield was 21 (IQR: 15–32 days) after minimally invasive gastrectomy and 16 (IQR: 11–24 days) after open gastrectomy.

## Discussion

In this nationwide cohort study concerning patients who underwent acute surgery for esophageal and gastric cancer, short-term postoperative and oncological outcomes of minimally invasive resections were comparable to open resections..

Previous studies have shown the safety and feasibility of minimally invasive surgery for esophagogastric cancer in the elective setting [3–12]. For esophageal cancer, minimally invasive esophagectomy resulted in a shorter hospital stay, higher lymph node yield, similar radical resection rate and postoperative pulmonary complications, but higher reintervention rates in several population-based studies [5–8]. Due to the low number of acute esophagectomies, it was unfortunately not possible in this study to perform the analyses that would be required to reproduce these results for minimally invasive esophagectomy in the acute setting. For gastric cancer, minimally invasive gastrectomy in the elective setting was deemed to be safe and feasible regarding overall postoperative morbidity and mortality rates, as well as short-term oncological outcomes, and resulted in decreased wound complications and a shorter hospital stay compared to open gastrectomy [10, 12]. The current study of minimally invasive gastrectomy in the acute setting also demonstrated comparable oncological outcomes to the open approach, as well as a potentially decreased median length of hospital stay. However, the current study demonstrated a higher conversion rate of minimally invasive gastrectomies in the acute setting (23% versus 0.9% [12], 3.5% [11] and 10% [10] in the elective setting), as well as a potentially increased percentage of patients that underwent a reintervention after minimally invasive gastrectomy (23% versus 14% in the acute setting and 0.4% versus 0.4% [11], 1.2% versus 1.5% [12] and 17% versus 16% [10] in the elective setting for minimally invasive gastrectomy versus open gastrectomy, respectively). Unfortunately, no clear explanation for this difference could be deduced from the causes for the re-interventions as available in the data.

In order to correctly interpret the current results, it must be noted that esophagogastric cancer surgery has been centralized since 2011 in the Netherlands. This is one of the main reasons that 84% of the elective esophagectomies and 40% of the elective gastrectomies are performed by minimally invasive techniques in the recent years [6, 9]. A similar increase has been seen for the use of minimally invasive techniques in the acute setting. Most surgeons in the Netherlands implemented these techniques after participating in a hands-on course on minimally invasive esophagectomy and gastrectomy, followed by several cases with an experienced surgeon present. The influence of centralization and learning curves on postoperative outcomes also seem to be important in for esophagogastric surgery in the acute setting, as demonstrated by a recently published study from England [16]. High-volume cancer centers and surgeons are more experienced in managing patients following esophagectomy and gastrectomy, and have the appropriate infrastructure available [16]. As such, they might be better equipped to deliver consistent levels of high-quality outcomes for minimally invasive surgery in the acute setting as well which might explain the comparable outcomes for minimally invasive and open surgery in the current study.

Overall, acute surgical interventions for upper gastrointestinal malignancies are rare, accounting for only 2% of all surgical interventions for upper gastrointestinal malignancies in the Netherlands. This especially applies for surgical interventions for esophageal cancer, occurring approximately once yearly in a country with an incidence of approximately 2500 new esophageal cancer cases per year [17]. Interestingly, most patients who underwent an acute surgical intervention had prior histological confirmation of their cancer diagnosis, indicating that patients generally did not present with an acute symptom of an unknown malignancy. The majority of the gastric cancer cases had a cause for acute intervention other than bleeding or perforation. As the DUCA registry only registers the indication for acute intervention in 3 prespecified categories (bleeding, perforation and other), the frequency of obstruction as another important indication for acute surgical interventions in gastric cancer could not be researched. However, in patients with an obstruction, a gastroenterostomy or distal gastrectomy might have been more frequently performed. When the surgical procedures of patients without a specified reason for the surgical intervention are explored in more detail, it might indeed be that obstruction accounts for a large share of the not specified indications, as 30% of them underwent a gastroenterostomy (21/71) and 44% underwent a partial gastrectomy (31/71) (data not shown).

The population-based design with virtually complete inclusion of all patients in the Netherlands is a significant strength of the study, along with the prospective data collection and detailed information on strictly defined postoperative outcomes. However, there are some limitations of the current study that need to be addressed. First, the small numbers of patients in all groups precluded a direct comparison between minimally invasive and open surgery correcting for bias. This prevents firm conclusions to be drawn regarding the potentially observed benefits of minimally invasive compared to open surgery in the acute setting (e.g., the decreased median length of hospital stay, higher observed percentage of radical resections and increased median lymph node yield), as well as regarding the potential disadvantages of minimally invasive surgery (e.g., the increased percentage of re-interventions after minimally invasive gastrectomy compared to open gastrectomy). Second, the introduction of minimally invasive surgery for upper gastrointestinal malignancies occurred simultaneously with centralization of cancer care and the introduction of enhanced recovery after surgery (ERAS) programs in the Netherlands. It has been shown that centralization of surgery is associated with reduced complications and improved long-term survival [18–21] and that the use of ERAS programs protocols can reduce the length of hospital stay [22, 23]. As such, the observed outcomes in this study for both minimally invasive as open surgery are probably influenced by these factors. It must be further acknowledged as a limitation that, due to the privacy restrictions of the national database, individual hospital related factors such as postoperative management protocols and background experience in minimally invasive surgery might have influenced the results but were not available. This also applies to more detailed information regarding the reasons for surgical intervention, such as iatrogenic versus spontaneous perforations and the severity of the bleeding. Lastly, no long-term survival data are available for the patients in the DUCA registry.

Future research regarding this topic would benefit from an even larger cohort study that would allow for statistical analyses corrected for bias to compare minimally invasive surgery and open surgery for esophagogastric cancer in the acute setting. However, considering the rarity of these events, larger case series are probably difficult to find.

In conclusion, this nationwide cohort study demonstrates that acute surgical interventions for esophageal and gastric cancer are rare. For gastric cancer, minimally invasive surgery appears to be feasible and safe in the acute setting with at least comparable postoperative clinical and short-term oncological outcomes compared to open surgery, but a relatively high conversion rate.
